# Effectiveness of simulation in teaching immunization in nursing: a randomized clinical trial*

**DOI:** 10.1590/1518-8345.3147.3305

**Published:** 2020-06-19

**Authors:** Raphael Raniere de Oliveira Costa, Soraya Maria de Medeiros, José Carlos Amado Martins, Verónica Rita Dias Coutinho, Marília Souto de Araújo

**Affiliations:** 1 Universidade Federal do Rio Grande do Norte, Escola Multicampi de Ciências Médicas do Rio Grande do Norte, Caicó, RN, Brazil.; 2 Universidade Federal do Rio Grande do Norte, Departamento de Enfermagem, Natal, RN, Brazil.; 3 Escola Superior de Enfermagem de Coimbra, Unidade Científico-Pedagógica de Enfermagem Médico-Cirúrgica, Coimbra, Portugal.

**Keywords:** Simulation, Students, Nursing Education, Immunization, Education, Primary Health Care, Simulação, Estudantes, Educação em Enfermagem, Imunização, Educação, Atenção Primária à Saúde, Simulación, Estudiantes, Educación en Enfermería, Inmunización, Educación, Atención Primaria de Salud

## Abstract

**Objective:**

to evaluate the effectiveness of the clinical simulation on the cognitive performance of nursing students in adult immunization scenarios in the context of Primary Health Care.

**Method:**

a controlled and randomized pre-test and post-test clinical trial applied to random intervention and control groups. 34 undergraduate nursing students were selected and divided into two groups: classes with active participation of students and skills training (control); and classes with active participation of students, skills training, and clinical simulation (intervention).

**Results:**

the students in the intervention group performed better than those in the control group in the four assessments of cognitive performance, with statistical significance in the assessments of immediate (p=0.031) and late (1-20 days) (p=0.031) knowledge.

**Conclusion:**

from the simulation, students learn more in the short and medium terms. The information learned is retained for longer and the students are better prepared for the professional practice. Universal Trial Number: u1111-1195-2580

## Abstract (pt)

**Objetivo:**

avaliar a eficácia da simulação clínica no desempenho cognitivo de estudantes de enfermagem em cenários de imunização de adultos no contexto da Atenção Primária à Saúde.

**Método:**

ensaio clínico controlado e randomizado do tipo pré-teste e pós-teste aplicado a grupo intervenção e grupo controle aleatórios. Foram selecionados 34 estudantes de graduação em enfermagem, divididos em dois grupos: exposição dialogada e treino de habilidades (controle) e exposição dialogada, treino de habilidades e simulação clínica (intervenção).

**Resultados:**

os estudantes do grupo intervenção apresentaram melhores desempenhos, em relação ao grupo controle, nas quatro avaliações de desempenho cognitivo, com significância estatística nas avaliações de conhecimento imediato (p = 0,031) e tardio 1 - 20 dias (p = 0,031).

**Conclusão:**

a partir da simulação, os estudantes aprendem mais em curto e médio prazo. As informações aprendidas ficam retidas por mais tempo e os estudantes são mais bem preparados para a atuação profissional. Universal Trial Number: u1111-1195-2580

## Abstract (es)

**Objetivo:**

evaluar la eficacia de la simulación clínica en el desempeño cognitivo de estudiantes de enfermería en escenarios de inmunización de adultos en el contexto de la Atención Primaria de Salud.

**Método:**

ensayo clínico controlado y aleatorizado previo y posterior a la prueba aplicado a grupos de intervención y de control aleatorios. Se seleccionaron 34 estudiantes universitarios de enfermería y se los dividió en dos grupos: exposición dialogada y entrenamiento de habilidades (control) y exposición dialogada, entrenamiento de habilidades y simulación clínica (intervención).

**Resultados:**

los estudiantes del grupo de intervención presentaron un mejor desempeño en relación al grupo de control, en las cuatro evaluaciones de desempeño cognitivo, con significación estadística en las evaluaciones de conocimiento inmediato (p = 0,031) y tardío 1 - 20 días (p = 0,031).

**Conclusión:**

a partir de la simulación, los estudiantes aprenden más a corto y mediano plazo. La información aprendida se retiene durante más tiempo y los estudiantes están mejor preparados para el desempeño profesional. Universal Trial Number: u1111-1195-2580

## Introduction

In Brazil, the National Immunization Program (*Programa Nacional de Imunização*, PNI) is recognized for its great contribution in reducing the indicators of morbidity and mortality caused by vaccine-preventable diseases. In addition, in the international scenario, it is considered the program that offers the largest number of free immunobiologicals^(^ 1 ^)^.

It is important to highlight the important role of nurses to achieve the good results presented by the PNI since, within the scope of health units and services, these professionals contribute positively to processes that enable the immunization of the population. Some of these professionals’ attributions include the following: management of the vaccine room, training and coordination of the nursing staff for maintenance and administration of immunobiologicals, application of doses of immunobiologicals, appointments, planning and development of strategies to expand and enable access to immunobiologicals^(^ 1 ^)^.

In identifying and recognizing the duties and contribution of professional nurses in making the processes that lead to immunization feasible and effective, it is important to consider the need for qualification of the nursing students during graduation. For example, practical internships by themselves do not guarantee that the students will be prepared to deal with the different situations commonly encountered in the realities of the health services, especially in the vaccination room.

When entering the Basic Health Units (BHUs), for example, the newly-graduated nurse has the same responsibilities as the other nurses in these services. From this perspective, failures during their training can compromise the execution of tasks and culminate in unwanted performance and damage to the population’s health^(^ 2 ^)^.

Therefore, it is necessary to rethink topics such as the curriculum, the contents, and methodological approaches adopted in teaching in the context of Primary Health Care (PHC). In this way, the Pan American Health Organization (PAHO) and the World Health Organization (WHO) have encouraged countries to promote reforms and improvements in the education of health professionals focused on PHC, especially in the Latin American context^(^ 3 ^)^.

However, in the education context, more traditional strategies have still been used on a large scale^(^ 4 ^)^. In undergraduate nursing courses, for example, the strategies most used in PHC teaching are the following: workshops, teaching by projects, teaching by research, and internships^(^ 5 ^)^. Therefore, there is an urgent need to diversify the teaching and learning strategies used during the training of nurses. Furthermore, this learning needs to be significant in terms of applicability in the professional practice.

In this sense, simulation gains a prominent position when compared to other more traditional teaching and learning strategies since, in nursing education, simulation is identified as a teaching technique that uses technologies to replicate scenarios that simulate practice, in a controlled and realistic environment, where the student participates actively in the teaching and learning process in order to exhaustively practice, learn, reflect and evaluate products and processes^(^ 6 ^-^ 7 ^)^.

Corroborating this relevance and applicability in nursing education, a study involving 25 countries in Latin America and the Caribbean, in 246 nursing schools, recommends the development and implementation of clinical simulation experiences centered on PHC^(^ 8 ^)^. The same study also suggests the need to identify leaders in this area; however, difficulties such as the lack of funding, the deficit in simulation training for teachers and lack of support from funding institutions are some of the challenges for researches in this area^(^ 9 ^)^. Few nursing studies have compared the results of the students’ simulated clinical experiences with the results of the traditional clinical setting^(^ 10 ^)^.

Therefore, evaluating the effectiveness of different teaching and learning strategies - among them, simulation - in the teaching of PHC topics in nursing is shown to be timely and relevant. This study aimed to evaluate the effectiveness of the realistic simulation on the cognitive performance of nursing students in adult immunization scenarios in the context of the Primary Health Care.

## Method

This is a randomized pre-test and post-test clinical trial applied to randomized intervention and control groups. The study was conducted in a federal public university in the Northeast of Brazil, between May and June 2017.

It was approved by the Research Ethics Committee under protocol No. 1,958,827 and CAAE No. 64874817.3.0000.5537. After approval, it was registered on the Brazilian Clinical Trials Registry platform under protocol RBR-9sqr6b, UTN number: u1111-1195-2580.

The students participating in the study were regularly enrolled in the 5^th^ to 9^th^ semester of the Nursing Undergraduate course. The option to prioritize these students was due to their availability to take the course that made data collection possible. The initial non-probability convenience sample was of 58 students.

After consolidating the instrument of characterization of the population in an electronic spreadsheet, the data were forwarded to an independent statistician for randomization. In this procedure, the following variables were taken into account: gender, age, Academic Performance Index (Índice *de Rendimento Acadêmico*, IRA), work experience in the area of PHC, and diagnosis of the preferred representational system. The researcher had no interference in the designation of the individuals allocated to the two groups.

After designation, chi-square (X2) and Fisher’s exact tests were performed, for a significance level of 5%. To verify the normality of the data, the Shapiro-Wilk test was applied, also assigning a significance level of 5%. It was evidenced that age and IRA did not have a normal distribution, therefore, non-parametric tests were applied to these variables. Using the Mann-Whitney test, for a significance level of 5%, no evidence of statistical difference in age and IRA was found between the selected groups.

**Figure 1:**
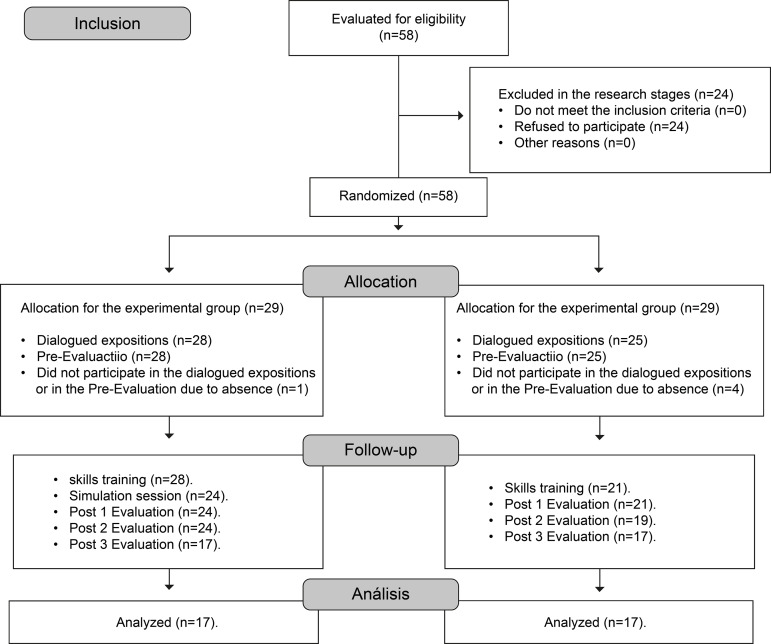
Follow-up diagram Adapted from CONSORT (2010)^(^ 11 ^)^

The following inclusion criteria were used: being a regularly enrolled undergraduate nursing student and having at least 75% attendance during the course offered. Students who were not present at the other times of intervention and application of the research instruments, scholarship students and collaborators who contributed to the execution of the study were excluded. After applying the inclusion criteria, the final sample consisted of 34 students, as detailed in Figure 1.

After randomization, the students participated in a 40-hour classroom course on adult immunization. The control group participated in the course in modality 1: (classes with active participation of students, and skills training); while the intervention group was directed to modality 2 (classes with active participation of students, skills training, and realistic simulation). Figure 2 details the strategies, learning objectives, and resources used in the interventions relevant to the training course that originated the data collection.

**Figure 2:** Teaching and learning strategies, learning objectives, resources used, and time of the interventions in the adult immunization training course. Natal, RN, Brazil, 2017

Teaching and learning strategy	Learning objective	Resources used	Time of the intervention
Lecture session	To know concepts related to the National Immunization Program; Cold chain; Vaccine room; Vaccination status of the adult.	Data show and presentation in Power Point.	8 hours
Skills training	Station 1 - Perform immuno-biological administration techniques. Station 2 - Know and identify routines and organization of the vaccine room. Season 3 - Schedule vaccine doses. Season 4 - Decision making regarding the vaccination status of adults.	Low-fidelity simulator for intramuscular and subcutaneous administration. Checklist dismembered with expectation of response. Short case with incomplete vaccination status. Short case with situations of contraindications and false contraindications.	20 minutes (each station)
Clinical simulation	Scenario 1 - Handle, from the point of view of immunization, a patient affected by a traumatic accident in the context of Primary Health Care. Scenario 2 - Schedule and administrate immunobiologicals. Scenario 3 - Recognize and make decisions in situations of contraindications in the administration of vaccines.	Scenario of a Basic Health Unit office. Standard patient (adult male with hand laceration). Scenario of a Basic Health Unit office. Standard patient (adult male). Scenario of a Basic Health Unit office. Standard patient (adult female holding breastfeeding infant on her lap).	50 minutes (per scenario)

The lecture classes were created from the contents provided and guided by the course syllabus. The lesson plan for each meeting was made available in advance, as well as a textbook with the references that served as the basis for each meeting. The references provided were taken from the PNI.

For skills training, checklist guides were made available. Four stations were set up in the nursing laboratory. On the occasion, the students were divided into small groups - 4 to 5 students - and took turns between the stations. After the consolidation of activities at each station, the researchers, a group of three nursing professors, provided *feedback* to the participating groups.

For the intervention group, the simulation scenarios were built from the instruments and references available in the literature from the models of the Tübingen University Hospital (TuPASS), Germany, and of the Anhembi Morumb University, Brazil^(^ 4 ^)^. In addition, the dimensions of the S.M.A.R.T structure (objectives, measurement of results, achievement of objectives, realism, and time) were taken into account^(^ 12 ^)^. The scenarios were tested and validated by specialists for appearance and content. The specialists consulted were the researchers of the project.

The scenarios were previously tested. For the simulations, the standard-patient tool was used, with actors trained to act and reproduce user behaviors in different situations and health care establishments^(^ 13 ^)^. The three scenarios were executed on the same day.

At the end of the simulations, the intervention group participated in the discussion and reflection, using the debriefing technique, stage in which all the students can discuss about the experienced scene. At that moment, the students had the opportunity to explore the scenarios experienced in order to help them consolidate the information acquired, identify and reflect on areas in which they could improve^(^ 14 ^)^. Each session lasted 30 minutes. With regard to the time of the session, it is important that it is not too long. It is recommended to be the double or triple of the scenario execution time^(^ 15 ^)^.

The researchers created a specific knowledge test about immunization of adults in the context of PHC, with 10 essay questions and an overall value of 10.0 points (1.0 per question). The test was applied in the intervention and control groups in four moments, namely: beginning of the course (Pre), immediately after the end of the course (Post 1), 20 days (Post 2) and 40 days (Post 3) after the course ended.

The tests were corrected by the researchers. The evaluation was guided by a solved question paper. The questions and corresponding expected answers were built from the contents and materials made available for the training course. The final score - in each evaluation - was established based on the mean assigned by two independent evaluators.

Data was analyzed in SPSS (Statistical Package for Social Sciences), version 24. For the characterization of the socio-demographic profile and evaluation of the course, descriptive statistics were used. In the analysis of cognitive performances, the Mann-Whitney test was used, for a significance level of 5%.

## Results

Most of the students who participated in the study were female (79.6%) and young adults. The most frequent age group was between 21 and 23 years old, with a mean of 22.3 years old (maximum of 34 and minimum of 18).

Regarding cognitive performance, Table 1 shows the values of the previous, immediate and late (20 and 40 days) evaluations. The intervention group (with simulation) had the best performance in all the evaluations, with an initial mean of 3.38 (maximum of 7.40 and minimum of 0.50) and a final mean of 6.55 (maximum of 9.00 and minimum of 3.00).

**Table 1:** Previous, immediate and late (Post 1 and Post 2) performances of the students in the control and intervention groups in the cognitive assessment test. Natal, RN, Brazil, 2017

	CG* (n=17)					IG^†^ (n=17)				
	Mean	SD^‡^	Median	Max^§^	Min^||^	Mean	SD^‡^	Median	Max^§^	Min^||^
Pre	3.35	4.22	2.80	3.80	0.90	3.38	2.23	2.80	7.40	0.50
Post 1	5.04	1.16	5.40	7.20	2.90	6.07	1.47	6.30	8.40	3.10
Post 2	5.55	1.10	5.70	7.60	3.00	6.35	1.25	6.60	8.10	3.70
Post 3	6.01	1.14	5.80	7.90	4.00	6.55	1.71	6.80	9.00	3.00

* CG = Control Group;

† IG = Intervention Group;

‡ SD = Standard Deviation;

§ Max = Maximum;

|| Min = Minimum

Although with lower performances, the students in the control group also showed an improvement during the four assessments, with an initial mean of 3.35 and a final one of 6.01. Both groups obtained increasing rates of performance in the short, medium and long terms.

The students in the intervention group (IG) had a better performance compared to the control group (CG) in the Post 1 (p-value = 0.031) and Post 2 (p-value = 0.031) assessments. This result suggests that, with the simulation, the students learn more in the short term and that the information learned is retained for longer.

No statistical significance was found in the previous (Pre) (p-value = 0.586) and Post 3 (p-value=0.231) assessments. Table 2 shows the mean values of cognitive performance in the four assessments of the CG and IG and the statistical significance from the Mann-Whitney’s U test.

**Table 2:** Mean cognitive performance (previous, immediate and Post 1 and Post 2) of the students in the control and intervention groups, and statistical significance. Natal, RN, Brazil, 2017

	Pre		Post 1		Post 2		Post 3	
	CG*	IG^†^	CG*	IG^†^	CG*	IG^†^	CG*	IG^†^
Mean	3.35	3.38	5.04	6.07	5.55	6.35	6.01	6.55
Mann-Whitney's U	128.000		82.500		82.500		109.000	
Z^‡^	-0.569		-2.138		-2.139		-1.223	
p-value^§^	0.586		0.031		0.031		0.231	

* CG = Control Group;

† IG = Intervention Group;

‡ Z = Z test;

§ Mann-Whitney's test

## Discussion

The study evaluated the effectiveness of the clinical simulation in the cognitive performance of nursing students in adult immunization scenarios in the context of PHC. It is known that Nursing has essential roles to guarantee the processes related to immunization, such as management of the vaccine room, organization and disposal of materials and supplies, conservation of immunobiologicals, and the nursing conduct^(^ 16 ^)^.

Although the relevance and contribution of the nursing professionals in the context of immunization are recognized, nursing errors are recurrent, such as the Adverse Events Following Immunization (AEFIs). Reports of these events after immunization are considered relevant worldwide^(^ 16 ^)^.

A Brazilian study that analyzed the occurrence of AEFIs due to immunization errors showed a significant increase in cases over a period of ten years. Thus, a disturbing scenario is observed since this type of error, linked to the nursing practice, can be avoidable^(^ 17 ^)^. This result raises concern as errors can interfere with the population’s confidence and, consequently, in the control of vaccine-preventable diseases^(^ 16 ^-^ 17 ^)^.

It is known that the PNI is the largest immunization program in the world. In this perspective, the offer and expansion of the number of immunobiologicals, the countless vaccination teams, the inadequate practices of conservation and administration of doses, and the constant updates in immunization schedules can contribute to errors^(^ 17 ^)^.

In this perspective, it is urgent to think about actions that promote safety and quality in immunization. Thus, thinking about teaching and learning strategies that promote meaningful learning is relevant and current^(^ 17 ^-^ 18 ^)^.

Several studies indicate strategies for improving safety in the scope of immunization, such as the use of protocols^(^ 15 ^)^ and improving the education of both^(^ 18 ^)^ students and professionals through continuing education^(^ 19 ^)^.

In the educational field, educational approaches that consider practical experiences have a substantial character. Thus, it is essential to rethink nursing education, especially when it comes to revisiting old assumptions, such as that the student’s learning is mainly related to the amount of information received from the teacher. The students are deemed to build their own cognitive structures and, from their interaction with the environment, to consolidate their knowledge^(^ 20 ^)^.

In this way, it is understood that learning becomes significant when there are relations, built by the students, between previous knowledge and new knowledge. Meanwhile, it is considered that when these relationships occur, there is effective, consolidated, and lasting learning^(^ 21 ^)^.

Significant learning is identified by the student when the acquired knowledge has applicability in the work practice^(^ 22 ^)^. Thus, the clinical simulation, as it has a realistic nuance, can be a teaching strategy to promote more consistent and significant knowledge^(^ 23 ^)^.

In this research, the students who participated in the training with the simulation had better performances - in the short and medium terms - when compared to those who were exposed to traditional teaching strategies.

Accordingly, different research studies present results similar to those found in this study. A research conducted with 58 undergraduate nursing students, which aimed to verify the effectiveness of the clinical simulation in sponge bath teaching, identified that the students who had an education associated with simulations had higher scores in the immediate and late (30 days after training with simulation) post-tests when compared to the rest^(^ 22 ^)^.

In contrast, a quasi-experiment conducted with 110 students in basic life support training evaluated the students’ knowledge and self-efficacy before and after the educational interventions. The results showed that there was no statistical significance in the acquisition and retention of knowledge between traditional teaching methods (Power Point presentation and demonstration) and high-fidelity simulation. However, the scores of the group with simulation were higher, both in terms of acquisition and of retention^(^ 24 ^)^.

A randomized, controlled and blind intervention study carried out with 34 nursing students evaluated the effectiveness of the clinical simulation in teaching how to evaluate deteriorating patients. It was observed that the experimental group had better scores in the post-test. In addition, the study identified the impacts of the clinical simulation and how effective it was compared to traditional teaching for developing skills to evaluate deteriorating patients^(^ 25 ^)^. The results of this research corroborate those found in the previous study by comparing and showing how effective the clinical simulation is compared to conventional teaching methods.

An experimental study, with pre- and post-tests, conducted with 85 nursing students, intended to evaluate the effect of a private simulation experiment on drug administration and identified that the simulation increased the student’s level of competence when compared to traditional teaching^(^ 26 ^)^.

Thus, traditional teaching methodologies, used occasionally, do not support quality education. In the context of nursing education, as science evolves, teaching and learning must be improved to keep up with the current health needs and changes^(^ 27 ^)^. In addition, the premise for having a quality training of nurses demands adequate and proper infrastructure, structured syllabus, and partnerships^(^ 28 ^)^.

When thinking about quality training and its requirements, one should consider the current job market, new technologies, current health demands, patient safety, and ethical issues^(^ 27 ^)^. To this end, it is necessary to use teaching and learning methodologies that consider these aspects, such as the clinical simulation, which is seen as a potential teaching and learning strategy as it is based on the aforementioned factors.

Regarding the stages of the simulation strategy and its potential for meaningful learning, the student’s participation in the debriefing stands out. In this phase, students can be guided in identifying gaps in the performance of tasks and their improvement^(^ 29 ^-^ 30 ^)^. In summary, there is the possibility of reflecting on the actions and on improving learning for future situations^(^ 31 ^)^.

Compared to other teaching strategies, the clinical simulation has the advantage of promoting organized and planned knowledge, where the student is the active participant in this process. Combined with the simulation, this structure has a greater impact on the students compared to feedback^(^ 32 ^)^. Questioning, exchange of experiences and knowledge about the experiences, the performance, the strategies for improving the actions and the transposition of this experience into work practice are part of this teaching and learning strategy.

High-quality simulated learning has the potential to be transformative, to engage emotions and to enable students to be directly involved in activities that reflect experiences in the workplace^(^ 33 ^)^.

The use of simulation has been increasingly present in nursing education^(^ 34 ^)^. Several research studies report benefits and acquisition of skills and abilities such as empathy, articulation between theory and practice, reduction of errors, decision making, leadership development, improvement in the health service processes and even increase in the levels of satisfaction, autonomy and self-confidence^(^ 35 ^-^ 41 ^)^.

Some benefits of the simulation include flexibility of access - without depending on the scheduling of days and hours in the clinical practice; a safe setting, both physically and psychologically, so that students can develop skills and make mistakes without causing damage to the users; the prior use of technologies that exist in the real practice; and the possibility of experiencing situations that are not commonly found in the practice - due to the impossibility of diagnoses, patient discharge, and/or lack of opportunities^(^ 42 ^)^.

Given the recognition of the possibilities and benefits of using the simulation in the context of teaching and learning in health and nursing, the WHO recommends its use in this context^(^ 43 ^)^.

Most of the conducted and disseminated studies that address the use of the simulation in nursing education are focused on urgencies and clinical emergencies. The research studies on high-fidelity simulation and the use of standard-patients in nursing and in the context of PHC is still incipient^(^ 44 ^)^. In this sense, better designed studies contribute to the production of evidence, to the expansion of the applicability of its use, and to the improvement of the quality of vocational training^(^ 45 ^)^.

While recognizing the relevance of training skills related to immunization practices, both in undergraduate courses and for the work practice, these trainings are not usually available in adequate formats in the educational institutions.

By comparing the effectiveness of the simulation with traditional teaching methods, this study contributes to reduce the gap in the national and international literature. In addition, evidence of the effectiveness of this strategy in nursing education can provide theoretical support for discussions about improvements in the educational process and the insertion of this strategy in the syllabus of nursing students.

It also contributes to the advancement of knowledge in the area of simulation and in the nursing field, as it uses an experimental design with a very considerable follow-up period. In researching this area of knowledge, most studies that use this design and are found in the literature have relatively short follow-up times.

One of the limitations of the study was the scarcity in the literature regarding research studies that could serve as a comparison and that mentioned the use of the simulation in the context of PHC - specifically about immunization. Another limitation was the number of losses during follow-up. As it originated from an extension course with several meetings and activities, the students had difficulties in reconciling it with other mandatory academic activities.

## Conclusion

The students in the experimental group had better performances in the assessment of cognitive knowledge in all the tests when compared to the students in the control group. There was statistical significance in the Post 1 (p = 0.031) - immediately after the intervention - and Post 2 (p = 0.031) - 20 days after the intervention. Thus, in this study, the clinical simulation promoted a more effective learning (from the point of view of cognitive performance) among nursing students in adult immunization scenarios in the context of PHC .
